# Interplay Between Optimal Ventilation and Gas Transport in a Model of the Human Lung

**DOI:** 10.3389/fphys.2019.00488

**Published:** 2019-04-26

**Authors:** Frédérique Noël, Benjamin Mauroy

**Affiliations:** ^1^Laboratoire JA Dieudonné, UMR CNRS 7351, Université Côte d'Azur, Nice, France; ^2^VADER Center, Université Côte d'Azur, Nice, France

**Keywords:** lung, control of ventilation, power dissipation, optimization, oxygen and carbon dioxide transport, convection, diffusion, mathematical model

## Abstract

Ventilation is at the origin of a spending of energy coming from air circulation in the bronchial tree and from the mechanical resistance of the tissue to motion. Both amplitude and frequency of ventilation are submitted to a trade-off related to this energy, but they are also submitted to a constraint linked to the function of the lung: to transport enough oxygen and carbon dioxide in order to respect metabolism needs. We propose a model for oxygen and carbon dioxide transport in the lung that accounts for the core physical phenomena: lung's tree-like geometry, transport of gas by convection and diffusion, exchanges with blood and a sinusoidal ventilation. Then we optimize the power dissipated by the ventilation of our model relatively to ventilation amplitude and period under gas flow constraints. Our model is able to predict physiological ventilation properties and brings interesting insights on the robustness of different regimes. Hence, at rest, the power dissipated depends very little on the period near the optimal value. Whereas, at strong exercise any shift from the optimal has dramatic effect on the power. These results are fully coherent with the physiological observation and raises the question: how the control of ventilation could select for the optimal configuration? Also, interesting insights about pathologies affecting ventilation could be derived, and our model might give insights on therapeutical responses, with specific breathing strategies or for better driving mechanical ventilation.

## 1. Introduction

The respiratory system's function consists in supplying the body with oxygen and in removing carbon dioxide. Each part of the respiratory system is regulated according to the others. Hence, the control of ventilation is coupled to the control of heart rate, so that both lung ventilation and blood circulation are coordinated in order to cope with body needs. Regulation is performed through inputs from sensors, amongst which sensors to oxygen and carbon dioxide partial pressure in blood play a crucial role. As a consequence, partial pressure in oxygen and carbon dioxide in blood are strongly regulated in mammals (Weibel, 1984). While oxygen directly affects the cells' access to energy, carbon dioxide drives blood pH, whose allowed range for healthy cellular function is tight (Madshus, 1988). The transfer of these two species between lung and blood are driven by the amount of blood flow in pulmonary capillaries, the gradient of the partial pressure between alveoli and capillaries, the blood/alveoli membrane characteristics and the properties of the ventilation cycle. In order for ventilation to handle blood homeostasy, it has to drag from blood a sufficient amount of carbon dioxide and to feed blood with sufficient amounts of oxygen.

Ventilation decomposes into inspiration and expiration. During inspiration, an amount of ambient air is internalized in the lung and fresh oxygenated air with low carbon dioxide is brought into the bronchi, closer to the exchange surface. From there, diffusion occurs downward the bronchial tree for oxygen, as blood acts as an oxygen pump, and upward the bronchial tree for carbon dioxide, as blood acts as a carbon dioxide source. The internalized volume of air is then expelled during expiration and replaced by new fresh air during the next inspiration. The control of ventilation is based on the regulation of the volume of air that is internalized (ventilation amplitude) and the frequency at which this volume of air is renewed (ventilation frequency) with the aim to keep oxygen and carbon dioxide partial pressure constant in blood.

**Figure 1:**
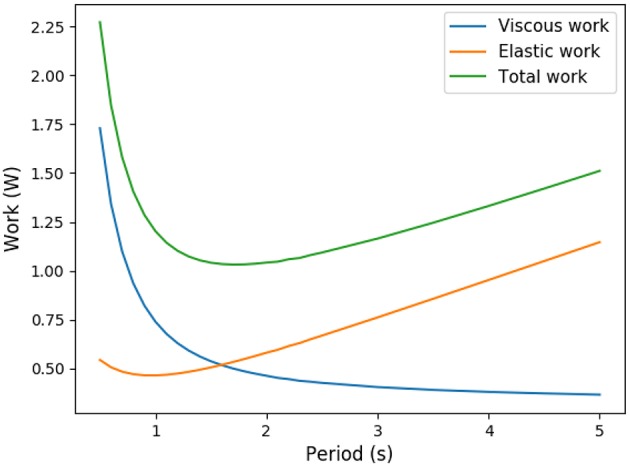
Trade-off between elastic energy stored in the tissue and viscous energy dissipated in the air circulation (exercise regime, computed from our model).

Ventilation amplitudes and frequencies are stereotypic in human and mammals (West et al., 1997). To explain this stereotypy, scenarios based on energy minimization have been proposed in the past (Johnson, 2007). The amount of power spent for ventilation comes from two main physical phenomena: the dissipation due to air circulation in the bronchi, related to the hydrodynamic resistance of the lung, and to the elastic power stored in lung's tissue. For the same amplitude, a higher frequency will increase the elastic power, while for the same frequency, a high amplitude will increase the dissipation in the bronchi. This raises the trade-off shown on Figure 1 and, using optimization theory, optimal ventilation frequencies and amplitudes have been predicted in the literature (Otis et al., 1950; Mead, 1960; Johnson, 2007). These predictions are not far from physiological values (Mead, 1960), predicting a frequency of 13 breaths per minute at rest that compares well with the typical 12 breaths per minute measured in Feher (2017). They consider energetic aspects only and assume that oxygen and carbon dioxide flows are maintained whatever the ventilation properties, neglecting the effect of ventilation on lung's function itself. Different ventilation amplitudes or frequencies cannot in general lead to the same gas flows at exchange surface, and ventilation's regulation aims at respecting blood homeostasy. This leads to a set of more detailed models that include links between ventilation, blood gas regulations and control (Grodins et al., 1967; Saunders et al., 1980; Cheng et al., 2010) or even neural controls (Ben-Tal and Tawhai, 2013). These models are built on several interacting compartments mimicking the behavior of the respiratory organs and are based on large sets of parameters. These models are powerful and allow to fit many physiological responses but each model compartment is a black box with a set of parameters. Typically, the lung's model is based on a single compartment with a one way air flow in Grodins et al. model (Grodins et al., 1967), on three compartments in Saunders et al. model (Saunders et al., 1980) and in a set of about 30 parameters in Cheng et al. model (Cheng et al., 2010). Although it allows to fit precisely physiological responses, such black boxes approaches do not allow to understand the physical phenomena linking the lung's properties and the ventilation characteristics. Moreover, it makes the identification of the core phenomena driving lung's ventilation very difficult.

In this work, we propose a transversal approach for this problem, by developing a model based on the core physical phenomena involved in lung's ventilation and function (Weibel, 1984). Our goal is to explain qualitatively the ventilation characteristics observed at different metabolic regimes. Two main approaches have been proposed in the literature to model gas transport in the lung, one based on effective diffusion in porous media (Koulich et al., 1999; Kuwahara et al., 2009; Berger et al., 2016) with the benefit of working with an homogeneous material mimicking the distal lung, and one based on tree-like structures (Kitaoka et al., 2000; Sapoval et al., 2002) with the benefit of having a detailed description of gas transport. Our model is based on this last approach, with the hypotheses that the lung is a bifurcating tree (Weibel, 1984; Mauroy et al., 2004) connecting ambient air to an exchange surface. This tree is ventilated with a sinusoidal pattern for air flow. The tree structure is a core aspect to understand how transport in the lung decomposes into convective and diffusive transport (Weibel, 1984; Mauroy et al., 2003; Sapoval and Filoche, 2008), and how these two phenomena connect with ventilation amplitude and frequency. We assume that blood homeostasy is the result of a specified constant amount of oxygen and carbon dioxide flow at the level of the exchange surface. Our model's ventilation is controlled by a virtual regulation system that would always select for the optimal ventilation with minimal mechanical power expenditure. Our study aims at comparing how our model's ventilation can compare to lung's physiological ventilation.

We show that the system built from these hypotheses can predict ventilation amplitudes and frequencies compatible with the physiology at different functional regimes and allows to understand finely and quantitatively the lung's inner dynamics of gas transport.

In the next section, section 2, we will describe our model for oxygen and carbon dioxide transport in the lung and the energy we propose to optimize. In section 3, we present and analyse the optimal ventilation in three emblematic cases: exercise, altitude and changes in lung's geometry. In section 4, we discuss the model's predictions and limitations, before concluding in section 5 on model's perspectives.

## 2. Modeling Oxygen and Carbon Dioxide Transport in an Idealized Lung

### 2.1. Hypotheses

Our model is based on the set of following assumptions:

- The lung is modeled as a tree with symmetric bifurcations.
- The lung is divided in two parts: the convective tree (17 first generations) and the acini (6 last generations).
- The size of the branches in the convective tree decreases at each bifurcation with a constant ratio.
- All branches in the acini are of the same size
- Oxygen and carbon dioxide are transported with air by convection and by diffusion. Exchange with blood is accounted for in the acini with a reaction term.
- Air fluid mechanics is incompressible and reduces to mass conservation in the bifurcations.
- Gas concentration at the trachea inlet is equal to that in the air.
- In blood, oxygen is mainly linked to hemoglobin and carbon dioxide is mainly linked to bicarbonate ions.

All these assumptions are detailed in the next sections.

### 2.2. Model for the Lung Geometry

The geometrical model mimicking the bronchial tree is based on a symmetric dichotomic bifurcating tree (Mauroy et al., 2004). A generation corresponds to branches with the same number of bifurcations up to the root of the tree that mimics the trachea. The tree is divided into two distinct parts (Weibel, 1984): the first 17 generations form the conductive tree and the last six generations form the acini where exchanges with blood occur.

#### 2.2.1. Convective Tree

To account for the core geometrical properties of the lung, we assume that the size of the branches in the conductive tree decreases from one generation to the next with a ratio 0 < *h* < 1 (Weibel, 1984; Mauroy et al., 2004; Karamaoun et al., 2018):

$$\begin{array}{c} l_{i+1}=l_{i}h\Rightarrow l_{i}=l_{0}h^{i},\\ r_{i+1}=r_{i}h\Rightarrow r_{i}=r_{0}h^{i}, \end{array}$$

with *l*_*i*_ the length and *r*_*i*_ the radius of a generation *i* branch (*i* ∈ [[0, *G*]], with *G* = 16). We can express the lumen area *S*_*i*_ of a branch in generation *i* with the radius, $S_{i}=\pi r_{i}^{2}$. Consequently, the area of one branch decreases with the generation, $S_{i}=h^{2i}S_{0}$. The number of branches in a generation is however increasing with the generation index, $n_{i}=2^{i}$. The volumetric flow rate in a branch from generation *i* is the product of the fluid velocity *u*_*i*_ and the lumen area of the branch *S*_*i*_. As air compressibility effects in the lung are considered small during forced expiration (Elad et al., 1989), the ventilation regimes studied in the frame of this work allow us to assume air as an incompressible fluid. Consequently, mass conservation between a branch in generation *i* and its two daughters in generation *i* + 1 leads to a scaling on the mean air velocity *u*_*i*_ in bronchi,

$$\begin{array}{l} u_{i+1}S_{i+1}=\frac{u_{i}S_{i}}{2}\;\Rightarrow \;u_{i}={\left(\frac{1}{2h^{2}}\right)}^{i}u_{0} \end{array}$$

#### 2.2.2. Acini

The last six generations mimic the acini. We assume that the size of the branches in acini remains constant (*h* = 1) in accordance with Weibel (1984). For all generations, we assume that the radius *r*_*A*_ and the length *l*_*A*_ are respectively equal to the radius and the length of the last generation branches of the conductive tree. Likewise the lumen area of branches remains constant and is $S_{A}=h^{2G}S_{0}$. Finally we can deduce the mean air velocity in a branch in the acini,

$$\begin{array}{l} u_{A,i+1}=\frac{u_{A,i}}{2}\;\Rightarrow \;u_{A,i}=\frac{u_{A,0}}{2^{i}} \end{array}$$

where *u*_*A,i*_ is the mean fluid velocity in a branch in generation *i* ∈ [[0, *H*]]. Based on our hypotheses for the conductive tree, we have $u_{A,0}={\left(\frac{1}{2h^{2}}\right)}^{G}\frac{u_{0}}{2}$.

### 2.3. Oxygen and Carbon Dioxide Transport in the Lung

The transport of oxygen and carbon dioxide in the bronchial tree is driven by three main phenomena: convection, diffusion and exchange with the acini walls. In the proximal conductive branches, the transport of the two species is mostly made through convection as transport velocity is higher than diffusive velocity. In the distal conductive branches, gas conductive velocities have decreased enough, and diffusion is dominating the species' transport thanks to the exchange occurring on the acini walls. We will describe the fluid motion along the axis of the bronchi, using a one dimensional model in bronchi. As bronchi and fluid properties are the same in all branches from the same generation, equations of transport are the same for each branch in a same generation.

#### 2.3.1. Convective Tree

The transport dynamics of the partial pressure of oxygen and carbon dioxide in a single branch of the conductive tree is given by the following convection and diffusion equation,

(1) $$\begin{array}{l} \frac{\partial P_{i}}{\partial t}-\underset{\text{diffusion}}{\underset{︸}{D\frac{\partial^{2}P_{i}}{\partial x^{2}}}}+\underset{\text{convection}}{\underset{︸}{u_{i}\left(t\right)\frac{\partial P_{i}}{\partial x}}}=0\;\text{ for }x\in \left[0,l_{i}\right]. \end{array}$$

*u*_*i*_(*t*) is the velocity of the fluid (m · s^−1^), *D* is the diffusion coefficient in air of the species considered (m^2^ · s^−1^) and *P*_*i*_ is the *O*_2_ or *CO*_2_ partial pressure (mmHg).

#### 2.3.2. Acini

Oxygen and carbon dioxide transport and exchange in the acinus consists in a convection and diffusion equation with a reaction term mimicking the exchanges with blood. In a branch belonging to an acinus, partial pressure of oxygen or carbon dioxide checks

(2) $$\begin{array}{l} \frac{\partial P_{A,i}}{\partial t}-\underset{\text{diffusion}}{\underset{︸}{D\frac{\partial^{2}P_{A,i}}{\partial x^{2}}}}+\underset{\text{convection}}{\underset{︸}{u_{A,i}\left(t\right)\frac{\partial P_{A,i}}{\partial x}}}+\underset{\text{exchange with blood}}{\underset{︸}{\beta \left(P_{A,i}-P_{blood}\right)}}=0\text{ for }\\ \;x\in \left[0,l_{A}\right], \end{array}$$

where *P*_*A,i*_ is the partial pressure of the gas (mmHg), *P*_*blood*_ is the partial pressure of the gas in the blood (mmHg), and finally β is an exchange coefficient. We can express β as follow (Weibel, 1984),

(3) $$\begin{array}{l} \beta =\frac{2k}{r_{A}}\alpha =\frac{2k}{r_{A}}\frac{D_{gas,H_{2}O}\sigma_{gas,H_{2}O}}{\tau }, \end{array}$$

*k* is the ratio relating partial pressure of the gas to its concentration in water. α (mol · m^−2^ · s^−1^ · mmHg^−1^) represents the permeability of the alveolar membrane. The coefficient *D*_*gas*,*H*_2_*O*_ is the diffusion coefficient of the gas in water (m^2^ · s^−1^), σ_*gas*,*H*_2_*O*_ is the solubility coefficient (mol · m^−3^ · mmHg^−1^) of the gas in water and finally τ is the thickness (m) of the alveolar membrane.

#### 2.3.3. Physical Analysis of the Transport

Equations (1, 2) can be adimensionalized and bring forth three adimensional numbers:

$$\alpha_{i}=\frac{l_{i}^{2}}{DT}\;Pe_{i}=\frac{l_{i}u_{i}}{D}\;\gamma_{i}=\frac{\beta l_{i}^{2}}{D}$$

**Figure 2:**
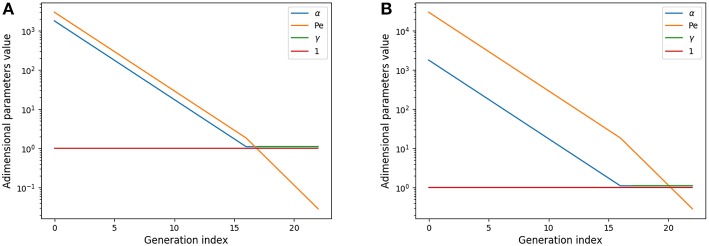
Adimensional numbers at rest **(A)** and exercise **(B)**. Results for oxygen. The case of carbon dioxide is very similar as diffusion coefficients of oxygen and carbon dioxide are similar. α is the relative amplitude of the transitory effects and of the diffusion; Peclet number *Pe* is the relative amplitude of the convection and of the diffusion; γ is the relative amplitude of the gas capture by blood and of diffusion.

α_*i*_ represents the relative amplitude of the transitory effects and of the diffusion; the Peclet number *Pe*_*i*_ represents the relative amplitude of the convection and of the diffusion; and γ_*i*_ represents the relative amplitude of the gas capture by blood and of diffusion and is meaningful only in the acini. These numbers are plotted on Figure 2A for rest and on Figure 2B for exercise.

These numbers show interesting insights on the behavior of gas transport. At rest, diffusion becomes dominant near the acini inlet only. Convection is dominant in the whole conductive tree. Transitory effects are slightly smaller than convection effects in the convective tree. Interestingly, in the acini, transport by diffusion, absorption by blood, and transitory effects are all similar in amplitude. At exercise, convection is dominant on every other phenomena down to the middle of the acini, where diffusion, absorption by blood and transitory effects become dominant and with similar amplitude. Diffusion and transitory effects are of the same amplitude because of the geometrical properties of the acini. The fact that absorption by blood is also of the same amplitude is the consequence of the value of $\frac{\beta l_{i}^{2}}{D}$ which is close to 1.

This analysis shows that all transport phenomena have to be included in the equations in order to reach satisfactory predictions for different metabolic regimes. Depending on these regime, the physics of transport and exchange with blood is driven by the three adimensional numbers previously defined.

#### 2.3.4. Bifurcations

The bronchi are connected together with bifurcations. We define a new variable *P*_*bif,i*_ which represents the mean gas partial pressure in a bifurcation of generation *i*. Mass conservation in the bifurcation leads to

(4) $$\begin{array}{l} V_{bif,i}\frac{\partial P_{bif,i}}{\partial t}=S_{i}\left(u_{i}\left(t\right)P_{i}\left(l_{i},t\right)-D\frac{\partial P_{i}\left(l_{i},t\right)}{\partial x}\right)-2S_{i+1}\\ \left(u_{i+1}\left(t\right)P_{i+1}\left(0,t\right)-D\frac{\partial P_{i+1}\left(0,t\right)}{\partial x}\right), \end{array}$$

with *V*_*bif,i*_ (m^3^) the volume of the bifurcation *i* approximated as follow

$$\begin{array}{l} V_{bif,i}=\pi \left(\frac{r_{i}^{3}}{2}+r_{i+1}^{3}\right). \end{array}$$

We approximated the volume of a bifurcation as the volume of three tubular extension of the three branches involved in the bifurcation: one with radius *r*_*i*_ and length *r*_*i*_/2 and two with radius *r*_*i*+1_ and length *r*_*i*+1_/2.

#### 2.3.5. Boundary and Initial Conditions

In order to close the system of equations, boundary conditions at both ends of the tree are needed. We assume *P*_0_(0, *t*) = *P*_*air*_ at the inlet of the root of the tree that models trachea. *P*_*air*_ is the partial pressure of the gas considered in the air. And for the end of the last generation of the acini, we use a flux boundary condition, based on the exchange laws previously defined,

$$\begin{array}{l} -D\frac{\partial P_{A,H}}{\partial x}\left(l_{A},t\right)=\alpha k\left(P_{A,H}\left(l_{A},t\right)-P_{blood}\right). \end{array}$$

Finally, an initial condition is needed. We suppose that at time *t* = 0, we have *u*_0_(0) = 0, $\frac{\partial P_{i}}{\partial t}\left(0\right)=0$ in the bronchial tree and *P*_*A,i*_ constant in the acinus. We also suppose that *P*_*bif,i*_ = *P*_*i*_(*l*_*i*_) = *P*_*i*+1_(0). With this hypotheses, we can compute the explicit solution of this system in the whole conductive tree :

$$\begin{array}{l} P_{i}\left(x\right)=P_{air}+\frac{P_{blood}-P_{air}}{\overset{N}{\underset{k=0}{\sum }}{\left(\frac{1}{2h}\right)}^{k}}\left(\overset{i-1}{\underset{k=0}{\sum }}{\left(\frac{1}{2h}\right)}^{k}+{\left(\frac{1}{2h}\right)}^{i}\frac{x}{l_{i}}\right). \end{array}$$

We solve this system with an implicit scheme in space using the language Julia. Given a ventilation pattern, we compute sufficient ventilation cycles to reach a periodic pressure pattern in time.

### 2.4. Blood Partial Pressures

#### 2.4.1. Modeling the Exchanges Between Alveolar Gas and Blood

In Equation (2), the term *P*_*blood*_ is actually dependent on time, space and gas species. Oxygen can be found in blood dissolved in the plasma and linked to hemoglobin. To compute *P*_*blood*,*O*_2__ for oxygen, we use the same formulation as in Felici (2003). Introducing *v*_*s*_ as blood velocity (m · s^−1^), we can relate *P*_*blood*,*O*_2__ with several physiological quantities,

$$\begin{array}{l} \alpha \left(P_{AO_{2}}-P_{blood,O_{2}}\right)=4Z_{0}\left(f\left(P_{blood,O_{2}}\right)-f\left(P_{aO2}\right)\right)v_{s}\\ \;+\sigma v_{s}\left(P_{blood,O_{2}}-P_{aO_{2}}\right). \end{array}$$

The first term in the right hand side represents the link to hemoglobin. The factor 4 corresponds to the fact that a molecule of hemoglobin can link 4 molecules of oxygen. *Z*_0_ represents the concentration of hemoglobin in the blood (mol · m^−3^). The function *f* is the oxyhemoglobin fraction at the equilibrium. This function is usually modeled using the Hill equation (Hill et al., 1936),

$$\begin{array}{l} f\left(x\right)=\frac{x^{2.6}}{x^{2.6}+26^{2.6}}. \end{array}$$

The second term in the right hand side of the equation represents oxygen solubility in the plasma. σ is the solubility coefficient (mol · m^−3^ · mmHg^−1^) of oxygen in blood and *P*_*aO*_2__ is the partial pressure of oxygen in pulmonary arterial blood.

Carbon dioxide can be dissolved in plasma, linked to hemoglobin and linked to bicarbonate ions. Similarly as oxygen, we can build an equilibrium law on carbon dioxide flow using (Sun et al., 2001),

$$\begin{array}{c} \alpha \left(P_{ACO_{2}}-P_{blood,CO_{2}}\right)=\\ \;\left(P_{blood,CO_{2}}-P_{aCO_{2}}\right)\sigma v_{s}\left(1+10^{\left(pH-pK\right)}\right)\\ \;\left(1-\frac{0.0289\;Z_{0}}{\left(3.352-0.456\;SO_{2}\right)\times \left(8.142-pH\right)}\right), \end{array}$$

with *v*_*s*_ the blood velocity (m · s^−1^), σ the solubility coefficient (mol · m^−3^ · mmHg^−1^) of carbon dioxide in blood, *pH* the blood pH, *pK* the dissociation coefficient of the chemical system $CO_{2}-HCO3^{-}$, *Z*_0_ the hemoglobin concentration (mol · m^−3^) and *SO*_2_ the oxygen-hemoglobin saturation (percents).

Practically, the partial pressure of oxygen *P*_*aO*_2__ and carbon dioxide *P*_*aCO*_2__ seen by the acini might be different to that of the pulmonary arterial circulation, as the history of the blood flowing in the acini wall is unknown. Blood could have already been in contact with acini air upstream. Consequently, we compute and use, instead of *P*_*aO*_2__ and *P*_*aCO*_2__, efficace partial pressures in oxygen ${\tilde{P}}_{aCO_{2}}$ and in carbon dioxide ${\tilde{P}}_{aCO_{2}}$ using a reference state and fitting the physiological parameters known for that reference state (rest).

#### 2.4.2. Efficace Partial Pressures Estimations

To compute the exchanges between alveolar air and blood, we need to estimate the efficace gas partial pressure in pulmonary arterial and venous blood (Feher, 2016). For low oxygenated blood (pulmonary arterial blood), *P*_*aO*_2__ = 40 mmHg and *P*_*aCO*_2__ = 47 mmHg. For oxygenated blood (pulmonary venous blood), we have *P*_*vO*_2__ = 100 mmHg and *P*_*vCO*_2__ = 40 mmHg.

Tidal volume (*V*_*T*_), mean air flow velocity in trachea (*u*_0_) and trachea cross-sections (*S*_0_) are related with:

$$V_{T}=\int_{0}^{T/2}u_{0,mean}S_{0}\text{d}t=0.5\cdot 10^{-3}.$$

The mean airflow velocity writes :

$$u_{0,mean}=\frac{2}{T}\int_{0}^{T/2}A\sin\left(\frac{2\pi }{T}t\right)\text{d}t=\frac{2A}{\pi }.$$

During rest ventilation, a human breathes around 12 times a minute, which corresponds to a period of *T* = 5 s; the tidal volume is about 0.5L = 0.5 · 10^−3^m^3^ (Feher, 2016). If we inject this expression in the tidal volume expression, we obtain *A* = 1m · s^−1^. With all these parameters our transport model gives us the amount of oxygen captured by blood (*VO*_2_) : 2.33 · 10^−4^mol · s^−1^ and the amount of carbon dioxide expelled from blood (*VCO*_2_): 1.06 · 10^−4^mol · s^−1^. These values are in the range of physiology which is around 1−2 · 10^−4^mol · s^−1^ (Jett et al., 1990).

The respiratory exchange ratio (RER) is defined as follow:

$$\text{RER}=\frac{VCO_{2}}{VO_{2}}.$$

**Figure 3:**
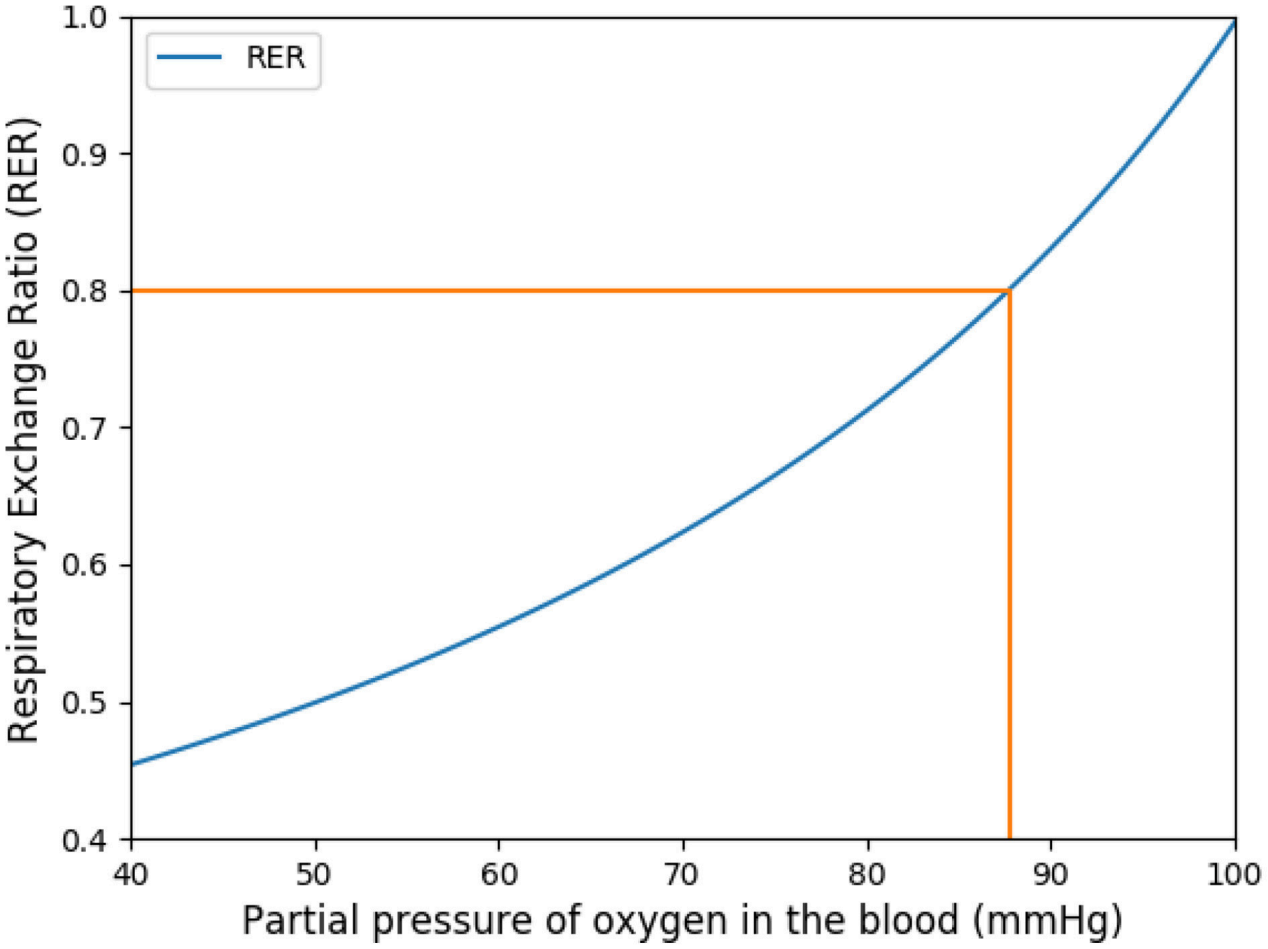
Respiratory exchange ratio in function of the partial pressure of the respiratory arterial blood.

**Figure 4:**
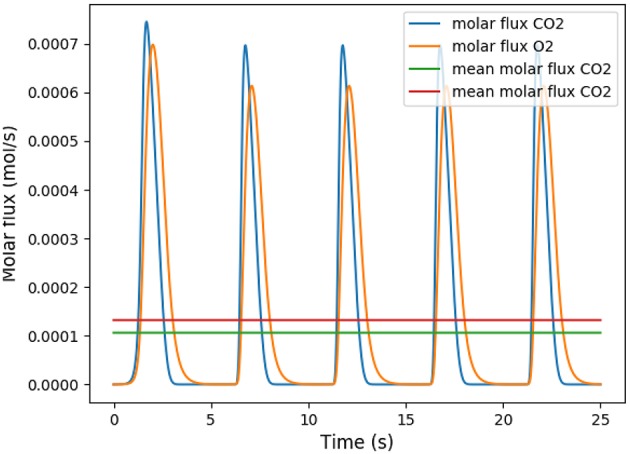
Number of molecules of oxygen and carbon dioxide per second during five respiratory cycle. The partial pressure in the arterial blood is set to *P*_*art*_ = 88 mmHg.

This coefficient is supposed to be between 0.7 and 1. Using in our model typical arterial and venous partial pressures in blood, this coefficient is predicted to be about 0.45. The physiological value at rest is however about 0.8 (Feher, 2016). In order to reach a correct value of RER, we have to account in our model for the fact that blood could have captured oxygen at its previous visited locations: we need to use an efficace partial pressures for arterial blood. We observed that in order to keep partial pressures in blood in the physiological ranges, it is sufficient to use an efficace oxygen partial pressure. As shown on Figure 3, the value ${\tilde{P}}_{art}=88$ mmHg allows to reach a correct RER. With this value, our model predicts at rest $VO_{2}=1.32\cdot 10^{-4}\text{mol}\cdot {\text{s}}^{-1}$ and $VCO_{2}=1.06\cdot 10^{-4}\text{mol}\cdot {\text{s}}^{-1}$ with a RER of 0.8, see Figure 4.

In order to validate the choice for efficace partial pressure, we did a perturbation analysis on the RER at rest and at exercise, see Table 1.

**Table 1:** Respiratory Exchange Ratio at rest with A the amplitude and T the period of the respiration.

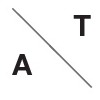	**4**	**5**	**6**
0.9	0.77	0.80	0.80
1	0.80	0.80	0.80
1.1	0.81	0.80	0.79

We have satisfactory little variation on RER when rest ventilation amplitude and frequency are perturbed and we remain in the physiological range.

During intense exercise, human can exhibit up to 40 breaths a minute, which represents a respiratory period *T* = 1.5 s. The tidal volume is then 2L = 2 · 10^−3^m^3^ (Feher, 2016). With similar computation as we did for rest, we compute an amplitude of 13m · s^−1^. The values of the RER during exercise predicted by our model are also shown on Table 2. They are fully compatible with the physiological data, as during exercise RER increases with the effort and comes close to 1 (Goedecke et al., 2000).

**Table 2:** Respiratory Exchange Ratio during exercise with A the amplitude and T the period of the respiration.

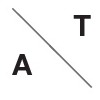	**1.4**	**1.5**	**1.6**
12	0.90	0.90	0.90
13	0.91	0.91	0.91
14	0.92	0.92	0.92

These last analyses show that our hypothesis to use an efficace partial pressure in oxygen for exchanges in our model leads to predictions fully compatible with expected physiological responses.

### 2.5. Choice of Physiological Parameters

Our model is based on a set of parameters that needs to be quantified from physiology. The parameters' list and values are shown on Table 3. The lung's geometry is defined from the radius (*r*_0_) of the root of the branch, mimicking the trachea, and from the homothetic ratio *h* that allow to compute the radius and lengths of all branches of the tree. The length of the branches is related on the diameter of branches using the ratio (Mauroy, 2004; Tawhai et al., 2004) $\frac{l_{0}}{2r_{0}}=3$, and we use *l*_0_ = 6*r*_0_. Although this value is not fully accurate for the main bronchi, it is a good approximation for the other branches. Since, the global behavior is mainly driven by the most numerous bronchi, extending the length over diameter ratio to all the branch of the tree is a reasonable approximation. Table 3 also gives quantitative values to blood flow velocity, alveolar membrane thickness, hemoglobin concentration in the blood, blood pH and finally pK coefficient, which drives the dissociation of the system $CO_{2}-HCO3^{-}$. These quantities are needed to describe properly the gas exchange between air and blood.

**Table 3:** Table of parameters for the environment.

**Parameters**	**Units**	**Values**
Radius of the trachea	m	10^−2^ (Mauroy, 2004)
Homothetic ratio (h)	Dimensionless	0.7937 (Mauroy, 2004)
Thickness of the alveolar membrane	m	1 · 10^−6^ (Felici, 2003)
Velocity of blood flow	m · s^−1^	5 · 10^−4^ (Felici, 2003)
Hemoglobin concentration in blood	mol · m^−3^	9.93 (Davis, 2017)
pH	Dimensionless	7.4 (Sun et al., 2001)
pK	Dimensionless	6.09072 (Sun et al., 2001)

Once the geometry of the lung defined, we can define the parameters linked to oxygen and carbon dioxide behavior, see Table 4. In order to solve Equations (1, 2), we need oxygen and carbon dioxide diffusion coefficients in air. The diffusion coefficient of the gas in water, the solubility coefficient of the gas in blood and the dimensionless Henry solubility allows to describe the gas exchanges between alveoli gas and blood. The dimensionless Henry solubility is the ratio between the aqueous-phase concentration of a gas and its gas-phase concentration. It can be computed as the product of the solubility coefficient in the blood, the gas constant and the temperature (Sander, 2015). The temperature in our model is fixed at 310.15 K (37^*o*^ Celsius).

**Table 4:** Table of parameters for the oxygen and the carbon dioxide.

**Parameters**	**Units**	**Oxygen**	**Carbon dioxide**
Diffusion coefficient in air	m^2^ · s^−1^	0.2 · 10^−4^ (Mauroy, 2004)	0.14 · 10^−4^ (Mauroy, 2004)
Diffusion coefficient in water	m^2^ · s^−1^	3.3 · 10^−9^ (Felici, 2003)	2.505 · 10^−9^ (Lu et al., 2013)
Solubility coefficient in the blood	mol · m^−3^ · mmHg^−1^	1.34 · 10^−3^ (Linder and Melby, 2018)	3.07 · 10^−2^ (Higgins, 2008)
Henry solubility	Dimensionless	2.592 · 10^−2^ (Sander, 2015)	0.594 (Sander, 2015)

### 2.6. Optimization Criterion: Minimizing the Energy Spent by the System

The power spent by the lung to bring air in contact with the exchange surface can be divided in two parts: a viscous part due to the resistance of the bronchial tree to air flow and a mechanical part due to the elasticity of the tissue. The viscous power is computed using the hydrodynamic resistance of the lung *R* ~ 2 · 10^5^Pa · m^−3^ · *s* (human)

$$P_{v}\left(A,T\right)=\frac{1}{T}\int_{0}^{\frac{T}{2}}Ru_{0}^{2}\left(t\right)S_{0}^{2}ds=R\frac{A^{2}S_{0}^{2}}{2},$$

where *A* is the amplitude and *T* the period of a respiratory cycle. The elastic power is based on the compliance *C* ~ 5 · 10^−7^m^3^ · Pa^−1^ (human). Compliance is commonly used to evaluate lung's elasticity, and relates lung's volumes with lung's pressures under static conditions. Compliance is a synthetic variable, mixing the effects of many biophysical phenomena occurring in the lung, such as tissue elasticity, surfactants' effects, etc. Compliance depends notably on lung's volume when deformation is high, as shown by the pressure-volume curves in Agostoni (1972). In this work, the compliance is assumed constant and we neglect the non linearities arising at large lung's deformations.

We assume elastic energy is brought during inspiration only, i.e., for $t\in \left[0,\frac{T}{2}\right]$, and dissipated completely during passive expiration, so

$$P_{e}\left(A,T\right)=\frac{1}{T}\int_{0}^{\frac{T}{2}}\frac{1}{C}V\left(t\right)\frac{dV}{dt}\left(t\right)=\frac{1}{C}\frac{A^{2}S_{0}^{2}T}{2\pi^{2}}$$

Consequently the total power dissipated is

(5) $$\begin{array}{l} P\left(A,T\right)=P_{e}\left(A,T\right)+P_{v}\left(A,T\right)=P_{e}\left(A,T\right)\left(1+\frac{\pi^{2}}{T}RC\right)\\ \;=\frac{A^{2}S_{0}^{2}T}{2\pi^{2}C}\left(1+\frac{\pi^{2}}{T}RC\right) \end{array}$$

The relative influence of each power source depends on the frequency *T*:

$$P\left(A,T\right)≃P_{e}\left(A,T\right)\left(1+\frac{1}{T}\right)$$

as π^2^*RC* ≃ 1. For normal human lung, *T* ≃ 5 *s* and the viscous part represents about 17% of the total power.

We aim at minimizing (A,T)→P(A,T) relatively to *A* and *T* with the constraint *f*(*A, T*) = *f*_*O*_2__(*A, T*) − *F* = 0 with *f*_*O*_2__(*A, T*) the flow of oxygen predicted with our model and *F* the desired oxygen flow.

**Figure 5:**
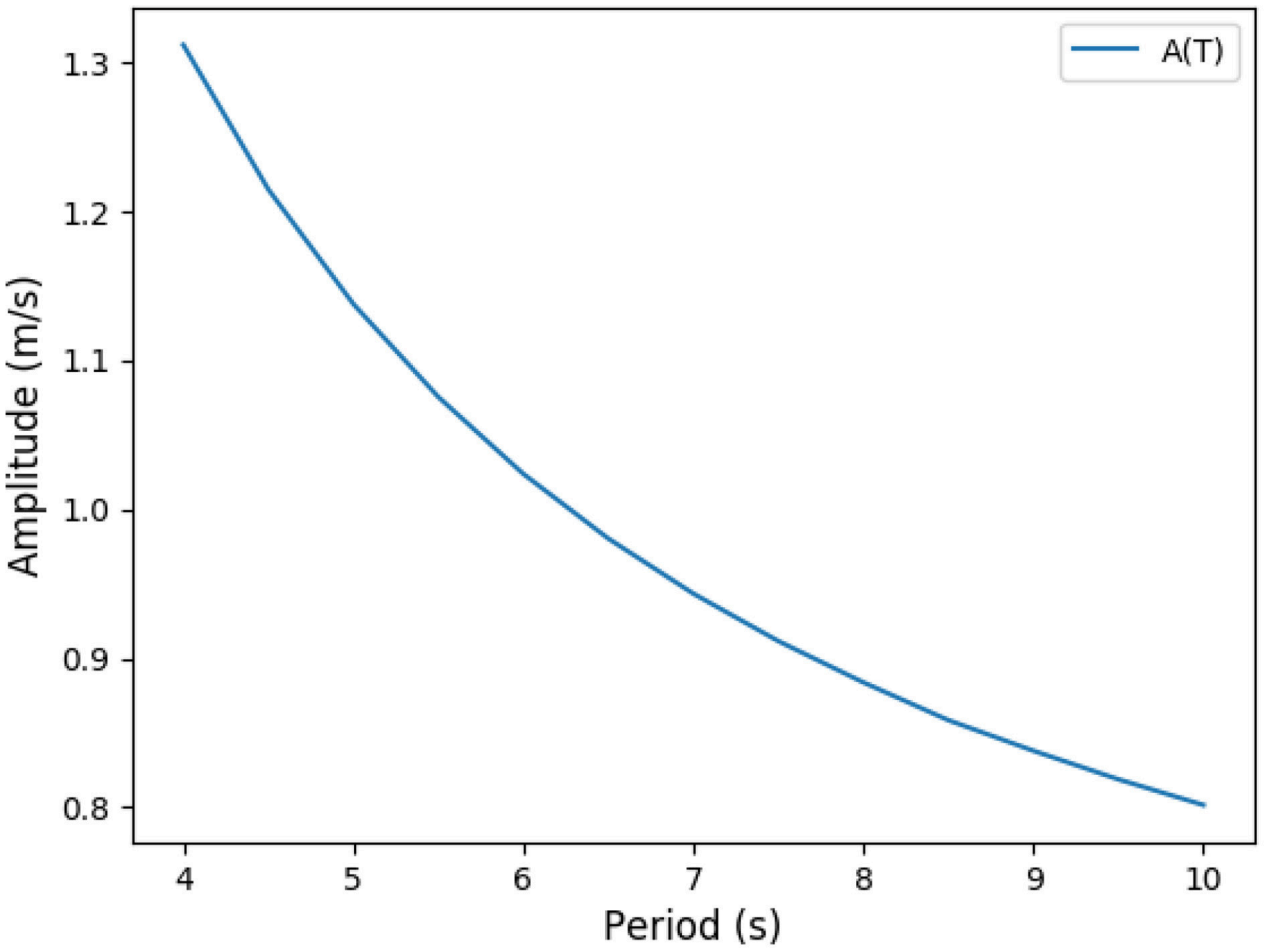
Predicted link between ventilation amplitude and period when the oxygen flow is constrained (rest regime).

Practically, ventilation period *T* and amplitude *A* can be linked through the constraint on the flow of oxygen to blood, in the form of a non linear function *T* → *A*(*T*). The non linear function is the result of the transport model of oxygen. For a given value for period, only one possible amplitude is possible in order to check the constraint. Carbon dioxide flow is the result of the model. Practically, the amplitude has to be high enough to bring the oxygen source and carbon dioxide drain close enough to the exchange surface so that diffusion is quick enough to drive the transport. Hence, for a high period, oxygen source can be further away from the exchange surface than for a short period. This behavior is shown on Figure 5 where the function *T* → *A*(*T*) is plotted. Consequently, with the oxygen flow constraint, the optimization problem is unidimensional and we search for the minimum of P or the zero of its derivative relatively to *T*

$$\frac{\partial P}{\partial T}\left(A\left(T\right),T\right)=\left(A^{\prime }\left(T\right)\left(1+\frac{T}{\pi^{2}RC}\right)+\frac{A\left(T\right)}{2\pi^{2}RC}\right)A\left(T\right)RS_{0}^{2}=0$$

consequently, we search for zero of $G\left(T\right)=A^{\prime }\left(T\right)\left(1+\frac{T}{\pi^{2}RC}\right)+\frac{A\left(T\right)}{2\pi^{2}RC}=0$. The function *T* → *A*(*T*) is computed thanks to the transport model in the lung, it is a positive decreasing function as shown on Figure 5. Interestingly, the optimal ventilation does not depend independently on the hydrodynamic resistance *R* and on the compliance *C*, as it depends on the product *RC* only. Hence, we are expecting the same behaviors for optimal ventilation relatively to changes in hydrodynamic resistance or changes in compliance.

## 3. Optimal Ventilation

Previous studies in the literature have been looking for optimal ventilation using modeling approaches, but they did not account for oxygen and carbon dioxide flows in the lung, or only in a basic way, without detailing the transport and exchanges of gas within a tree geometry and with detailed physics (Otis et al., 1950; Mead, 1960; Grodins et al., 1967; Saunders et al., 1980; Johnson, 2007; Cheng et al., 2010). In the model defined in the previous sections, we have a way to interpret the optimal ventilation and gas flows directly in term of the physical phenomena occurring in the lung. Our aim in this section is now to test our model's predictions against known physiological responses: exercise, altitude, and geometrical changes.

### 3.1. Impact of Physical Activity

**Figure 6:**
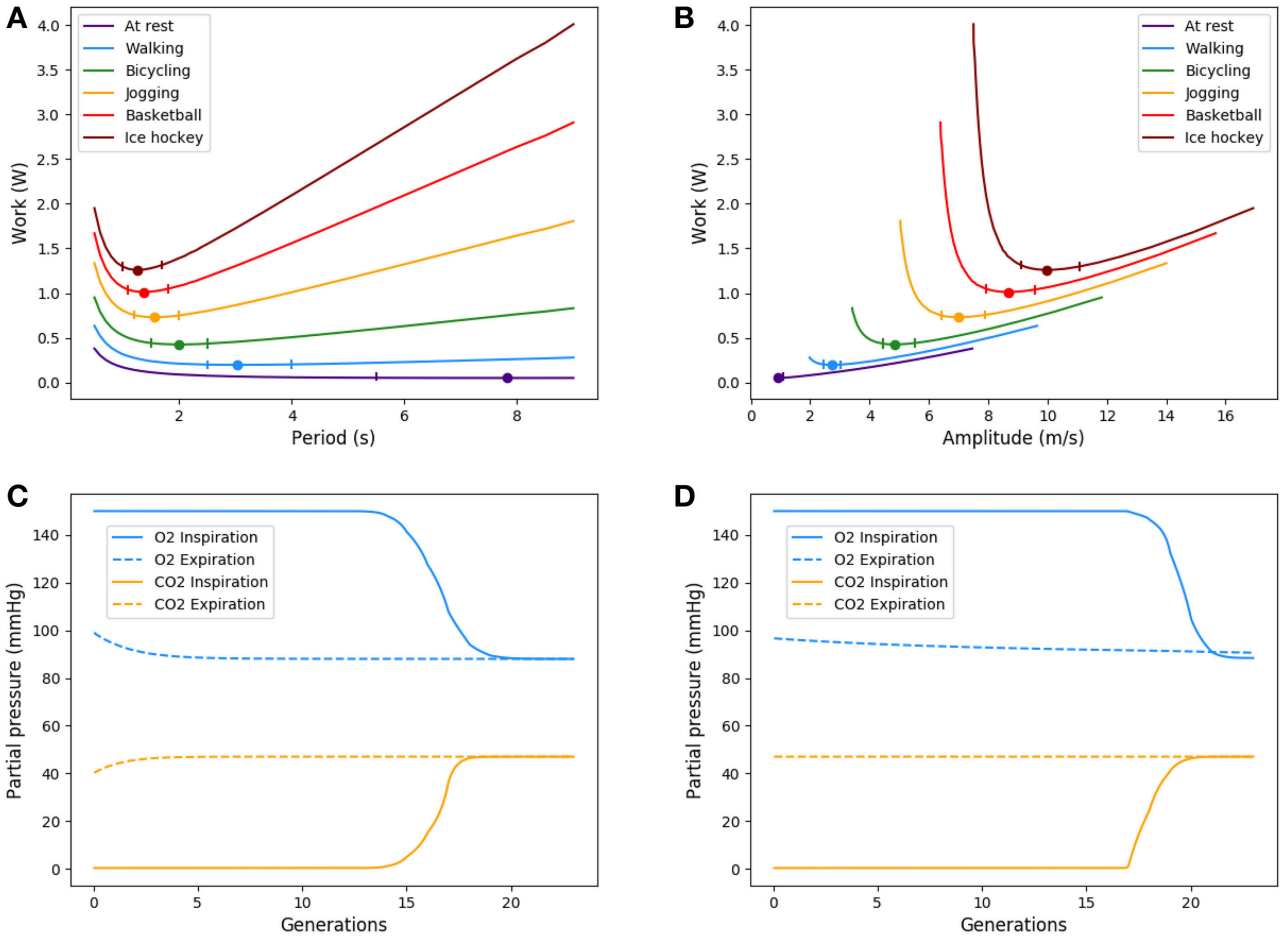
Up: Energy in function of amplitude and period for different types of exercise. Ticks represent +5% relative energy variations. Down: Partial pressures distributions, results are plotted for peak flows. At exercise, “fresh” air is pushed deeper in the lung. **(A)** Power vs. period, **(B)** Power vs. amplitude, **(C)** *O*_2_ and *CO*_2_ distribution in the tree at rest, and **(D)** *O*_2_ and *CO*_2_ distribution in the tree at exercise.

We ran the model for different amount of oxygen needs, mimicking physical activities with increasing intensity (Jett et al., 1990) as shown on Table 5. Our simulations are run for a 70 kg male person. Our results are plotted on Figure 6 and highlight two main interesting responses.

**Table 5:** Activities and the amount of oxygen consumed.

Walking for exercise (5 km/h)	$11.2\text{mL}O_{2}\cdot {\text{kg}}^{-1}\cdot {\text{min}}^{-1}$
Bicycling (15 km/h)	$20.65\text{mL}O_{2}\cdot {\text{kg}}^{-1}\cdot {\text{min}}^{-1}$
Jogging (9 km/h)	$30.80\text{mL}O_{2}\cdot {\text{kg}}^{-1}\cdot {\text{min}}^{-1}$
Basketball	$38.85\text{mL}O_{2}\cdot {\text{kg}}^{-1}\cdot {\text{min}}^{-1}$
Ice hockey	$45.15\text{mL}O_{2}\cdot {\text{kg}}^{-1}\cdot {\text{min}}^{-1}$

**Figure 7:**
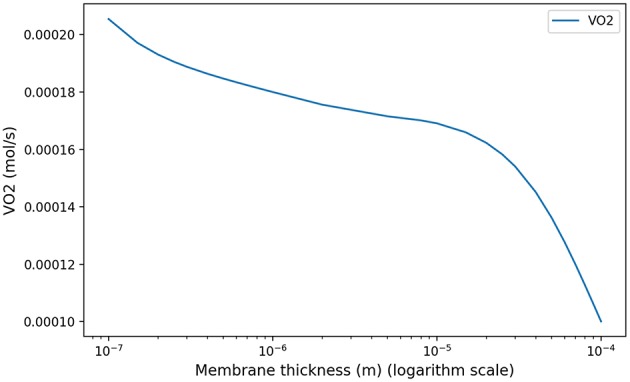
Model response in term of oxygen flow to an increase of membrane thickness at rest to mimic pulmonary oedema. As shown in Sapoval and Filoche (2008), screening effects allow to compensate the reduced efficiency of the exchange surface by mobilizing more surface, up to a point where the oxygen flow is crashing.

At low regimes, the model predicts relatively high optimal period *T* = 7.8 *s*, but the power dependence on the period is very low near the optimal. The model exhibits a robustness in term of period perturbation around the optimal. A 5% shift in the energy brings the period into a range between 5.5 s up to more than 10 s. This effect is due to the fact that, at low regimes, a low amplitude *A* is sufficient to perform an optimal ventilation. As the power depends on the period with the quantity *A*^2^*T*, if *A* is small then *A*^2^*T* remains small whatever reasonable values for period *T*. In term of oxygen transport, as soon as the oxygen source is deep enough in the tree, diffusion is able to reach easily the needed oxygen flow in the first acini generations because the flow is small and the deepest parts of the acini are not contributing substantially to oxygen flow: this effect is called acini screening (Sapoval and Filoche, 2008) and it is the highest at rest regime. The screening allows a reservoir of exchange surface for exercise and brings also a robustness to reduced efficiency of the exchange surface. Our model can mimic the physiological effects of pulmonary oedema by increasing the alveolo-capillary membrane thickness τ in Equation (3). As expected (Sapoval and Filoche, 2008), oxygen flow is not really affected up to a point where screening disappears and any subsequent increase of τ drastically reduces the oxygen flow, crashing suddenly the patient as shown on Figure 7.

When the exercise intensity increases, the power profiles as a function of the period become steeper and steeper and focus the optimal value within a tighter region. So our model predicts a finely tuned ventilation period at high regimes, in a way much more critical than at low regimes. An opposite behavior is observed for ventilation amplitude, but it is quantitatively lower. As amplitude increases, oxygen source goes deeper within the lung, entering the acini and increasing the exchange efficiency, but by thus draining more quickly oxygen from air. Renewing of the internalized air becomes more crucial to keep sufficient oxygen flow. A similar behavior occurs for carbon dioxide, but in the opposite direction. The increase in RER is due to a stronger response of carbon dioxide to a reduced screening as its diffusion coefficients are smaller than those of oxygen.

**Figure 8:**
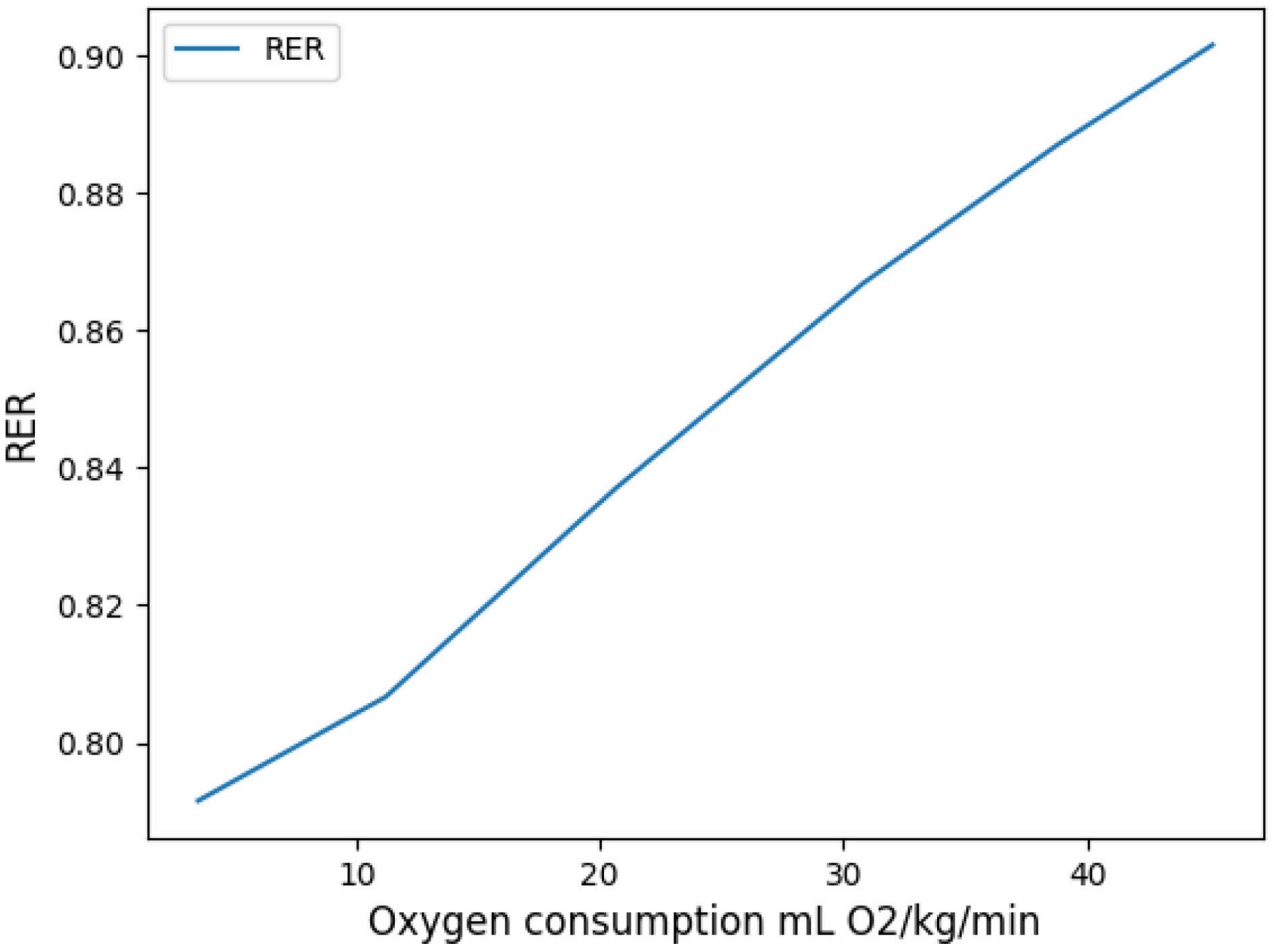
Respiratory Exchange Ratio as a function of oxygen flow.

Oxygen and carbon dioxide exchanges are tightly related in our model, but only oxygen exchanges are constrained. However, the model predicts a RER fully compatible with physiology: as expected it increases from 0.8 at rest up to 0.9 at high exercise, see Figure 8. These results are fully compatible with physiology (Goedecke et al., 2000). Moreover, the quantitative predictions in a range of variation of 5% of the power are in accordance with measured data. In the case of intense exercise (hockey), we observe that period reaches down to 1.5 s and amplitude about 10 m/s as commonly expected for intense exercise (Weibel, 1984).

### 3.2. Response to Altitude Hypoxia

Hypoxia is the consequence of an alteration of the flow between alveolar gas and blood. This is typically one of the response observed in high altitude (Peacock, 1998; Storz et al., 2010) where the flow is affected by the smaller pressure gradient at the air/blood interface. Hence we induced hypoxia by altering the partial pressure of oxygen in the ambient air while keeping the needed oxygen flow rate constant, see West (2004) and Table 6. In this framework, we can compare the predictions of our model with known physiological responses (Beall, 2006).

**Table 6:** Partial pressures in oxygen as a function of altitude in percents of sea-level partial pressure.

**Altitude (m)**	**Oxygen partial pressure**
	**(**%** of sea-level)**
0	100%
1,000	89%
2,000	79%
3,000	69%
4,000	60%

*Data from West (2004), sea-level oxygen partial pressure is 19.8 kPa*.

**Figure 9:**
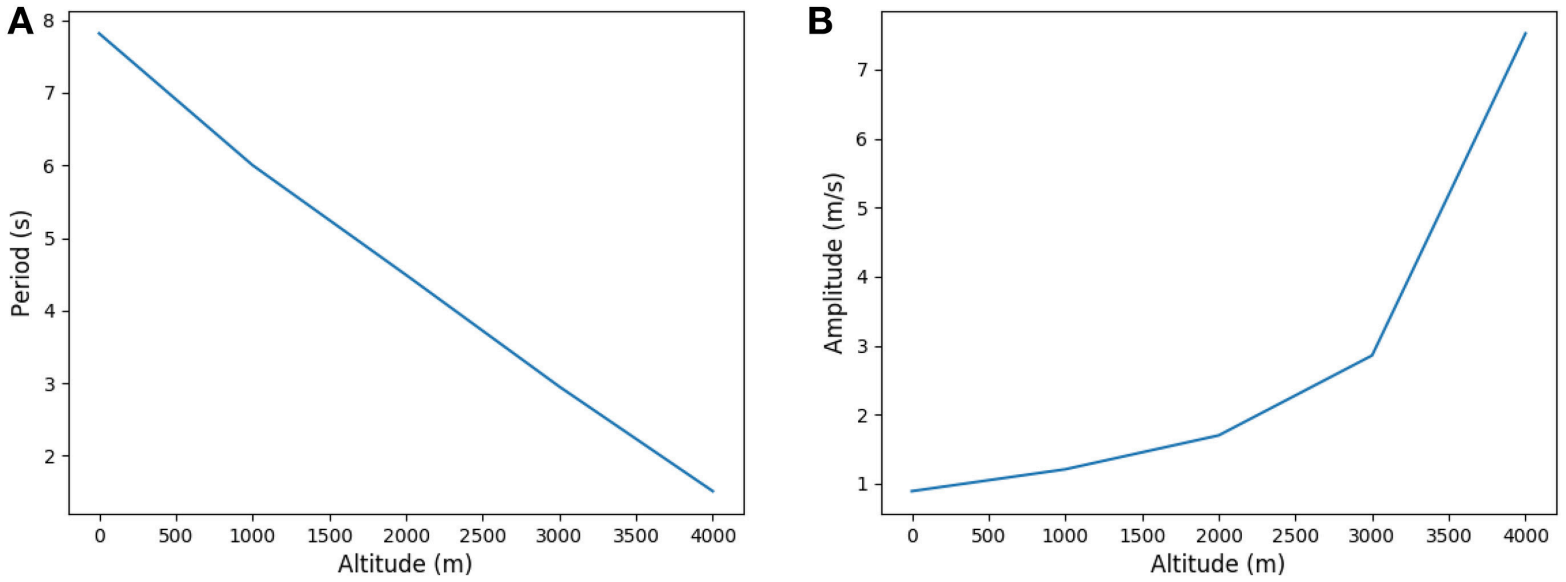
Period and amplitude in function of altitude. **(A)** Period and **(B)** Amplitude.

Our model predicts an increased ventilation in order to compensate the lower oxygen partial pressure, with higher amplitude and lower periods as shown on Figure 9. This allows to put the scarcer oxygen source deeper in the acinus and to benefit from a higher exchange surface to compensate for the lower oxygen gradient between alveolar gas and blood. The response is fully compatible with lung's physiology (Teppema and Dahan, 2010) and brings on the typical strategy of the lung to increase ventilation when gas exchanges are too low. When altitude is higher than 4,000 meters, the model is not anymore able to fulfill the oxygen flow constraint, implying that blood homeostasy is not anymore sustainable in our model.

### 3.3. Changes in Lung's Geometry and Mechanics

Optimal ventilation depends on the relative values of elastic and viscous powers. The power profile (but not its scaling) is driven by the quantity *RC* with *R* the hydrodynamic resistance of the lung and *C* its compliance, see Equation (5). Any change in one of these quantities affects the characteristics of the optimal ventilation. However, the reasons for a change in resistance and compliance are different. A change in hydrodynamic resistance is the consequence of a change in the geometry of the airways, while a change in compliance corresponds to a change in the mechanical response of the lung to volume changes, i.e., mainly related to lung's tissue properties. Consequently, a change of resistance in our model has to go with a change of geometry.

For increases of resistance that do not affect notably the amount of inhaled air volume, typically for local or proximal constrictions, we observed very low changes in the optimal configuration. The energy increases proportionally with the dissipative term. In this case, the relevant parameter for computing the optimal value is the product *RC* and this analysis also applies inversely to *C*.

**Figure 10:**
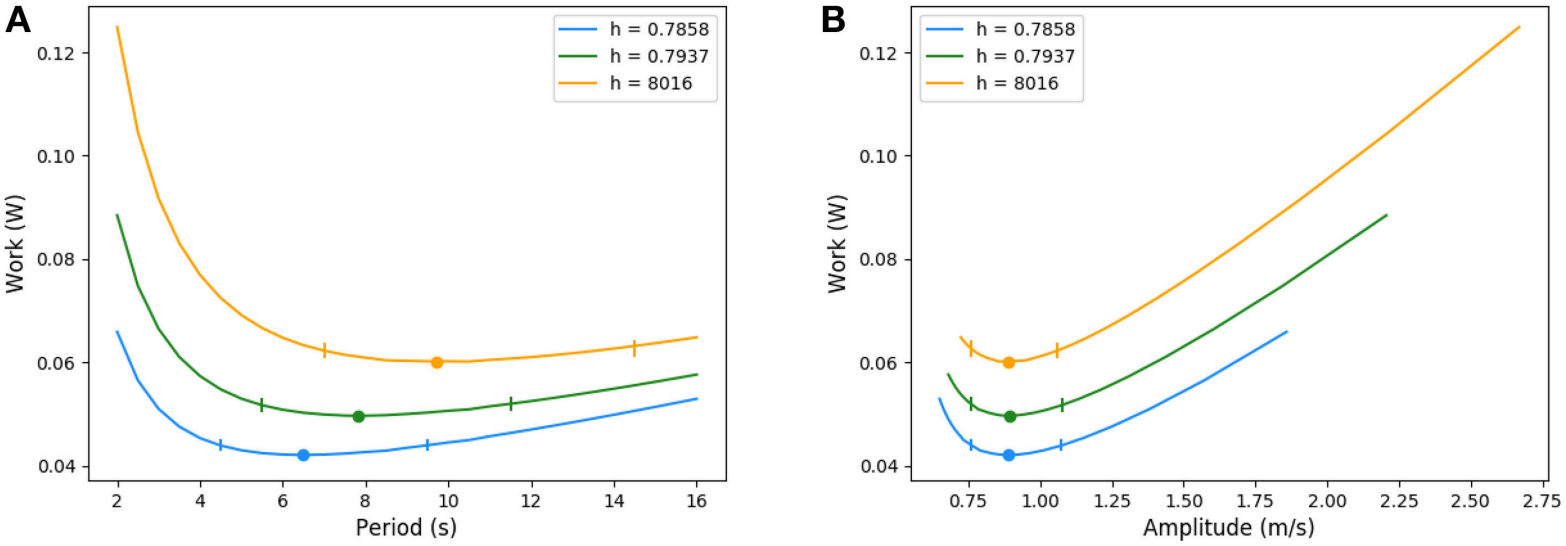
Energy in function of amplitude and period for different resistance values. Ticks represent +5% relative energy variations. **(A)** Period and **(B)** Amplitude.

For increases in resistance (see Appendix) that notably affect the amount of inhaled air, typically for smaller lungs, during chest compression or a global bronchi constrictions, the optimal ventilation changes significantly: as the amount of air to inhale is smaller, the optimal configuration keeps the same amplitude but lowers the period. Because the exchange surface is also smaller, diffusion explores deeper in the acini, leaving less reserve for exercise. In a whole, the total energy spent is smaller when resistance increase goes on with smaller lung volume. The energy curves are plotted on Figure 10, where resistance and geometrical changes have been related with the reduction ratio *h* between two successive generations: the amplitude of the velocity is almost the same for the three tree morphologies whereas the period decreases when the resistance increases. The highest resistance tested (about 30% larger than the reference state) corresponds to an optimal period of about 6.5 s, the reference resistance of about 7.8 s and the lowest resistance tested (about 30% less than the reference state) of about 9.8 s. In all these configurations, the power remains flat around the optimal value, the flat region is however shifting toward higher values when the lung's resistance decreases.

## 4. Discussion

### 4.1. Model's Physics and Application to the Lung

The optimal configuration for the ventilation of our lung's model is based on physical phenomena known to play a crucial role in the lung's function: gas convection, diffusion and exchange with blood (Weibel, 1984; Sapoval et al., 2002; Mauroy et al., 2003, 2004). The crucial point is that oxygen has to be pushed deep enough into the lung by air convection so diffusion can take over oxygen and carbon dioxide transport efficiently. Convection effects decrease exponentially down the lung because of its structure, so the location of the diffusion source is strongly related to the geometry itself. In parallel, the geometry of acini also plays a role on the efficiency of diffusion, with different responses depending on the metabolic regime involving the screening effect (Sapoval and Filoche, 2008). All these ingredients drive finely the flows of oxygen and of carbon dioxide.

Our model allows to analyse how the coupling of these phenomena could drive gas exchanges in a way that minimizes a total power, sum of the mechanical power (compliance) and of the dissipative power (hydrodynamic resistance) (Johnson, 2007). The cost in energy is due to the convective inspiratory phase and depends on the speed at which a volume of air is inhaled: if the speed is large, dissipative power is high, if the volume is large, mechanical power is large. The volume of air internalized and the geometry of the bronchial tree define at which depth the diffusive source will be localized in the lung. The source is then depleted in oxygen and filled with carbon dioxide so that for diffusion to remain efficient, the volume of air has to be renewed in time.

The control of ventilation in the lung is a complex dynamical and biophysical system that relies on many sensors' signals of different types (mechanical, chemical), with delays and complex integration. Many models have been proposed to mimic how the brain can control ventilation in accordance with metabolic regimes, see for example the reviews (Raux et al., 2007; Ben-Tal and Tawhai, 2013). Our approach here is not to study the control of ventilation itself, but instead to understand how the control might want to respond if the power is to be minimized, accounting for a detailed transport of oxygen and carbon dioxide. The transport model developed here accounts for the core physical phenomena, as discussed in the literature (Weibel, 1984). Hence we neglect several other secondary aspects that might play a role on the quantitative predictions, such as detailed model of lung's geometry (realistic branching asymmetry, proximal bronchi geometry, inhomogeneous ventilation, etc.), bronchi deformation, non sinusoidal ventilation signal, etc. Consequently, the results presented in this paper have to be interpreted in the frame of these limitations of our model.

### 4.2. Rest Regime

In the case of rest regime, our model predicts an optimal ventilation with an amplitude for air velocity in trachea of about 0.9 m/s, very close to the acknowledged physiological value of 1 m/s (Weibel, 1984), and a period for ventilation of about 7.8 s, higher than the physiological value of about 5 s (Weibel, 1984). Although the shift to physiological value is about 50%, the order of magnitude of our model's prediction is correct. Moreover, an analysis of the energy shape, plotted on Figure 10, shows that the power varies very little for a relatively wide range of periods near the optimal. If we allow an energy variation of up to 5% of its optimal value, the corresponding periods range from 5.5 s up to 12 s (see ticks on Figure 10). This shows that modulation of the period has very few consequences in term of energy. This suggests that rest easily allows our common lung's period adjustments, during talking, during temporary apnea or during practices based on long ventilation periods.

Our model is however not able to answer why the selected normal ventilation at rest stands in the lower values of the low energy plateau. A shift to lower period's values favors a lower mechanical energy and consequently higher amplitudes and dissipative energy. The answer might be linked to robustness criteria that are known to play an important role in strategies selection (Mauroy et al., 2004; Mauroy and Bokov, 2010; Florens et al., 2011; Vercken et al., 2012).

### 4.3. Exercise Regimes

Amongst the scenarios tested, three are considered intense: jogging, basketball, and ice hockey. At these regimes, measured data for the physiological period (Blackie et al., 1991) ranges between 1.3 and 2.2 s. The predictions of our model for these three scenarios range between 1.3 and 1.6 s, hence in good accordance with physiology. Measured data for physiological amplitude (Blackie et al., 1991) ranges between 7.6 and 21.8 m · s^−1^. The predictions of our model for amplitude are in this range, but more tightly packed toward the lower values, with amplitudes ranging between 7 and 10 m · s^−1^. A possible explanation for this packing toward the low values might be that our model does not account for the wide range of possible human physiology and body needs as our studies is based on one set of parameters only.

### 4.4. Control of Ventilation Favors Low Power Dissipation

The optimal way of ventilating our model in a wide range of regimes are very similar to the physiological responses of real lung's ventilation. This is more particularly true when the metabolic regimes increases: the energy profile becomes steeper and steeper near the optimal limiting the margin for adjusting both ventilation amplitude and period. This suggests that control of ventilation might have built-in energetic optimizer, either encoded in neural control, learnt or computed. Our model shows that their efficiency is crucial at high regimes, where the energetic costs are the larger. At high regimes, a shift from the optimal configuration is predicted by our model to be very costly and maintaining the regime might prove difficult shifted from the optimal. This behavior is fully compatible with the fact that control of ventilation is stronger at exercise, preventing even talking. This raises a fundamental question by which mechanisms the control of ventilation can adjust the ventilation so that it is nearly optimal ni term of power dissipation.

### 4.5. Hypoxic Response Might be a Non-optimal Response to a Resistance Increase of the Lung

In the case of an increase of lung's resistance due to a tighter geometry, the optimal strategy is to keep the same ventilation amplitude and to reduce the ventilation period. This leads to a ventilation process that is less energetic than a wider geometry. Although this sounds counter intuitive as higher hydrodynamic resistance is often correlated to tiredness and higher energy dissipation in the lung, our result shows that in the optimal strategy found, the resistance increase due to tighter bronchi is compensated by a volume of internalized air that is smaller (same amplitude, but for a lower time). This volume acts as a smaller oxygen source and ventilation period has to decrease for quicker renewal of the internalized air. In a whole, the mechanical energy gained with a smaller volume to move allows a decrease of the total energy. This behavior is however not the one expected from physiology, mainly because the effect of an increase of hydrodynamic resistance is first hypoxia, that induces ventilation control to use both a higher amplitude and a smaller period, as predicted by our model. The net result of hypoxia effects is an increase of the power spent for ventilation. So our analysis show that response to hypoxia might not be the best response for hydrodynamic resistance increase. Hypoxia is known to also be a typical maladaptive response to high altitude relocalization by activating processes that might be deleter for some individuals. Our results suggest that this might also be the case when mechanical characteristics of the lung are degrading.

## 5. Conclusion

We propose here a model of the gas transport in the human lung based on the core physical phenomena identified in the literature: tree structure of the lung, convective and diffusive transports of oxygen and carbon dioxide, exchange surface properties (size, thickness, etc.). We study the power dissipated (resistance to flow in the airways and mechanical energy stored in the tissue) for the ventilation of our model and how it depends on ventilation amplitude and period. We search for ventilation characteristics that minimize this power assuming the oxygen flow has to fit the need of the metabolism. The predictions of our model are very close to the physiology. At rest, the power depends weakly on the period and makes the system very robust to period adjustments; when the regime increases, the dependence in the period becomes steeper and steeper and any shift from the optimal value is critical in term of power dissipation. These results are fully compatible with the physiology, indicating that the phenomena included in our model might drive the main responses of control of ventilation in human. Since the power optimized in this study is based on physiological parameters routinely measured by physicians and physiologists, confirmation of the predictions of our model should be possible by experimental reconstruction of the power profiles.

Our study raises however two questions not answered by our model. First: why is the period of rest ventilation in human so stereotyped although power dependence is low? Second: What mechanisms make control of ventilation able to select at exercise for ventilation regimes that minimize power dissipation?

## Author Contributions

Analyses were performed by FN. Research topic was proposed by BM. Both authors contributed to modeling and writing of the paper.

### Conflict of Interest Statement

The authors declare that the research was conducted in the absence of any commercial or financial relationships that could be construed as a potential conflict of interest.

## Appendix

The power dissipated by viscous effects of air circulation is based on the hydrodynamic resistance of the lung *R*. Knowing the size reduction factor at bifurcation of the bronchi, we are able to compute the equivalent hydrodynamic resistance of the whole tree :

$$R=R_{0}\left[1+\overset{G}{\underset{p=1}{\sum }}\frac{1}{{\left(2h^{3}\right)}^{p}}+\overset{G+H}{\underset{p=G+1}{\sum }}\frac{1}{2^{p}h^{3G}}\right],$$

where *R*_0_ is the resistance of the branch of the first generation,

$$R_{0}=\frac{8\mu l_{0}}{\pi r_{0}^{4}},$$

with μ = 1.8 · 10^−5^Pa · *s* the viscosity of air. This expression neglects however the resistance of the bifurcations and the effects of inertia; using it, such as leads to an underestimation of lung's resistance.

Estimation of human lung's resistance have been extensively measured in the literature but its precise relation with the parameter *h* is not well known. In order to compute a value for the resistance with different *h*, we chose to compute the resistance with the previous formula and to determine the ratio of increase or decrease relatively to the reference value *h* = 0.7937. Then we multiply this ratio with the physiological value of the resistance in order to obtain a coherent, although approximative, change in the lung's hydrodynamic resistance.
